# Multifocal Oral Mucosal Melanoma with an Atypical Clinical Presentation

**DOI:** 10.3390/dj13090432

**Published:** 2025-09-18

**Authors:** Klaudia Podlińska, Monika Monist, Magdalena Sławińska, Wojciech Popowski

**Affiliations:** Department of Oral Surgery, Medical University of Warsaw, Binieckiego 6, 02-097 Warsaw, Poland; klaudiapodlinska@wum.edu.pl (K.P.); zcs1@wum.edu.pl (M.M.); magdalena.slawinska@wum.edu.pl (M.S.)

**Keywords:** oral mucosal melanoma, oral cancer, hyperplastic changes

## Abstract

**Background**: Oral mucosal melanoma (oral malignant melanoma—OMM) is a rare malignant neoplasm. It arises from the proliferation of atypical melanocytes—cells derived from the neural crest that produce melanin. Unlike cutaneous melanomas, which are etiologically linked to ultraviolet (UV) radiation exposure, the risk factors for mucosal melanomas remain poorly defined. According to the World Health Organization (WHO), these tumors predominantly affect older individuals, with peak incidence occurring in the seventh decade of life and are rarely observed in the first three decades. The primary treatment modality for patients with mucosal melanoma is radical surgical excision with clear margins. The 5-year overall survival rate for OMM ranges from 20% to 50%. **Case Presentation**: This article reports an atypical clinical manifestation of oral mucosal melanoma in a 99-year-old patient who presented to the Department of Oral Surgery at the University Dental Center, Medical University of Warsaw. The nonspecific clinical appearance did not initially suggest a melanocytic lesion. A definitive diagnosis was established through histopathological examination, which subsequently guided the treatment plan. **Conclusions**: This report highlights the necessity of performing microscopic evaluation even for lesions with a nonspecific or non-suspicious appearance, underlines the importance of regular dental check-ups, and stresses the need to strengthen oncological vigilance among dental practitioners.

## 1. Introduction

Primary oral mucosal melanoma (OMM) is an extremely rare tumor, accounting for 1–2% of all malignant tumors of the oral cavity and less than 1% of all melanomas. Its incidence increases with age, and most patients are over 60 years old, with a median age at diagnosis of 70 years [1]. It is more common in men [2,3,4] and is more prevalent among Japanese, African, and Native American populations than among Caucasians [5]. The etiology of oral mucosal melanoma remains unknown: there is no evidence that repeated trauma, chronic inflammation, or smoking play a role in its pathogenesis [5]. Ultraviolet radiation and associations with SMV, EBV, HPV, or HSV infections have been excluded as contributing factors to the incidence of OMM [1]. The only confirmed risk factor is a history of pre-existing mucosal melanin hyperpigmentation [5]. Most OMMs arise de novo from clinically normal mucosa, but approximately 30% of cases are preceded by oral pigmentation lasting for several months or even years [5,6,7].

Mucosal melanoma differs markedly from the more common cutaneous form. Clinically, mucosal lesions present late and lack the classic pigmented appearance, leading to delayed diagnosis and worse outcomes (often with 5-year survival under 20–25%). Biologically, these tumors exhibit low mutational burden with scarce BRAF (3–11%) mutations but more frequent KIT (22%) and NRAS (5–14%) aberrations, contrasting with the UV-driven mutation profiles seen in cutaneous melanoma [8,9]. Importantly, a substantial proportion of tumors—estimated at 40–55%—belong to the wild-type group, with no detectable mutations in the most frequently studied oncogenes [9,10,11]. Additionally, mutations in NF1, SF3B1, and SPRED1 genes further increase molecular heterogeneity, making therapeutic standardization difficult [9,10,11]. Beyond inter-tumoral variability, intra-tumoral heterogeneity has also been observed—mutational profiles may differ between tumor samples from the same patient. Given this molecular diversity, it is crucial to develop site-specific therapies and implement comprehensive molecular profiling to optimize treatment strategies—an approach that could improve therapeutic efficacy [9,12].

Surgical management is more challenging due to anatomical complexity, and therapeutic options—especially targeted or immunotherapies—are limited or less effective. Overall, mucosal melanoma is significantly more aggressive and carries a poorer prognosis than its cutaneous counterpart [13,14,15].

About 80% of primary melanomas located in the oral cavity occur in the keratinized mucosa of the hard palate, then the mucosa of the maxilla and mandible and the buccal mucosa. Cases of melanoma of the tongue or bottom of the mouth [7,16] and lips, soft palate, retromolar triangle [17] have also been described. Only 0.6–9.3% of patients with cutaneous melanoma will develop metastases in the mucosa of the upper gastrointestinal tract [18].

Amelanotic morphology accounts for approximately 10–25% of OMM cases and is associated with delayed diagnosis due to the absence of pigmentation [19]. True multifocality is only sporadically documented, typically described either as synchronous lesions or as local intraoral spread, and therefore remains a clinical rarity. Against this background, the current case is particularly notable for combining advanced age, gingival involvement, multifocal presentation, and amelanotic morphology. This constellation of features underlines both the diagnostic difficulty and the importance of maintaining a broad differential diagnosis when evaluating atypical oral lesions [19].

Several key prognostic factors are recognized in mucosal melanoma. Early-stage disease, thinner tumor thickness, and surgical resection are associated with improved overall survival [19,20]. Ulceration and nodal involvement similarly forecast poorer outcomes, and high mitotic activity along with vascular invasion have been linked to aggressive behavior and reduced survival. Anatomical location also plays a critical role: sinonasal tumors carry a particularly poor prognosis, especially in cases of large size or vascular invasion [20,21].

Metastases of melanoma to the oral cavity are rare, most commonly affecting the bones of the maxilla and mandible as well as the tongue, and their occurrence may indicate systemic spread of the disease [22]. At the time of oral mucosal melanoma diagnosis, regional lymph node metastases are present in approximately 25% of patients, while distant metastases occur in about 10% [5]. They are most frequently found in the lungs (54%), liver (35%), and bones (25%). In contrast, in the cutaneous form, metastases are most often detected in the skin (13–38%), lungs (18–36%), and lymph nodes (5–34%) [1].

In about two-thirds of cases, metastases arise through the lymphatic route, while in the remaining cases they spread via the hematogenous route [13].

The main treatment method for patients who develop mucosal melanoma is surgical treatment. Unfortunately, due to its sneaky evolution and late diagnosis at a usually advanced stage, the results of surgical treatment are not satisfactory.

The purpose of this report is to present an atypical clinical presentation of oral melanoma.

## 2. Case Presentation

In August 2024, a 99-year-old patient presented to the Department of Oral Surgery at the University Dental Center of the Medical University of Warsaw (WUM) with complaints of progressive gingival enlargement and recurrent bleeding during mastication localized in the regions of teeth 17–16 and 32–33. The patient was unable to determine the exact onset of these lesions and additionally reported inability to use dentures, which significantly impaired oral function. The patient’s oncological history was significant.

Four years earlier, the patient had been diagnosed with nodular malignant melanoma of the abdominal skin. The lesion was excised with tumor-free margins (Breslow thickness 4.5 mm, mitotic rate 8/mm^2^, no neurovascular invasion, stage pT4b AJCC). At that time, contrast-enhanced CT showed no additional lesions or suspicious lymphadenopathy, and the patient was placed under regular oncological follow-up. One year after the primary excision, local recurrence occurred in the abdominal scar, presenting as two nodular foci. Both were surgically re-excised with histologically negative margins. Since then, serial CT and ultrasound imaging demonstrated no evidence of metastases.

**Figure 1:**
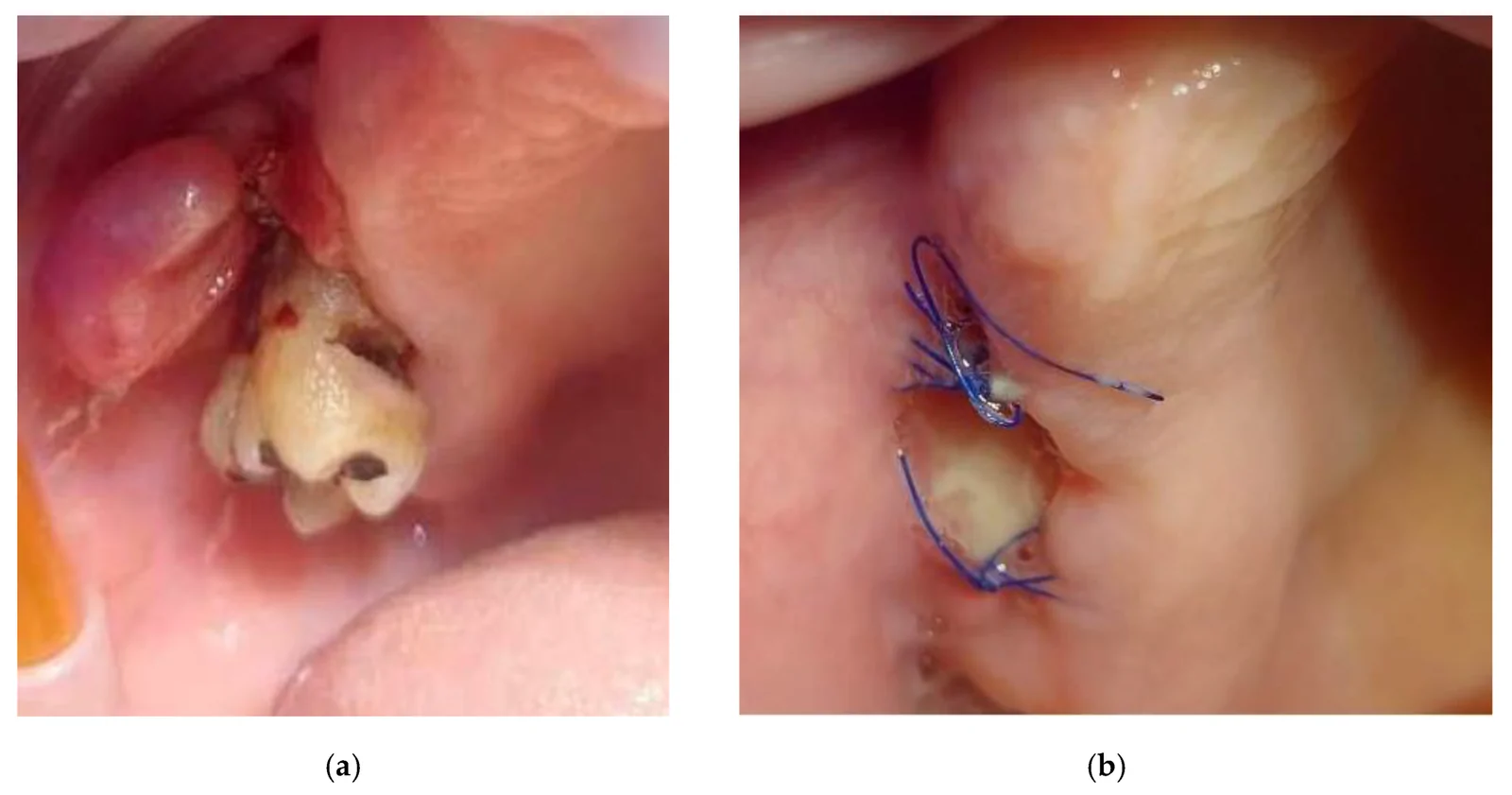
(**a**) Gingival mass in the area of tooth 16. (**b**) One week following surgical excision of a maxillary tumor.

**Figure 2:**
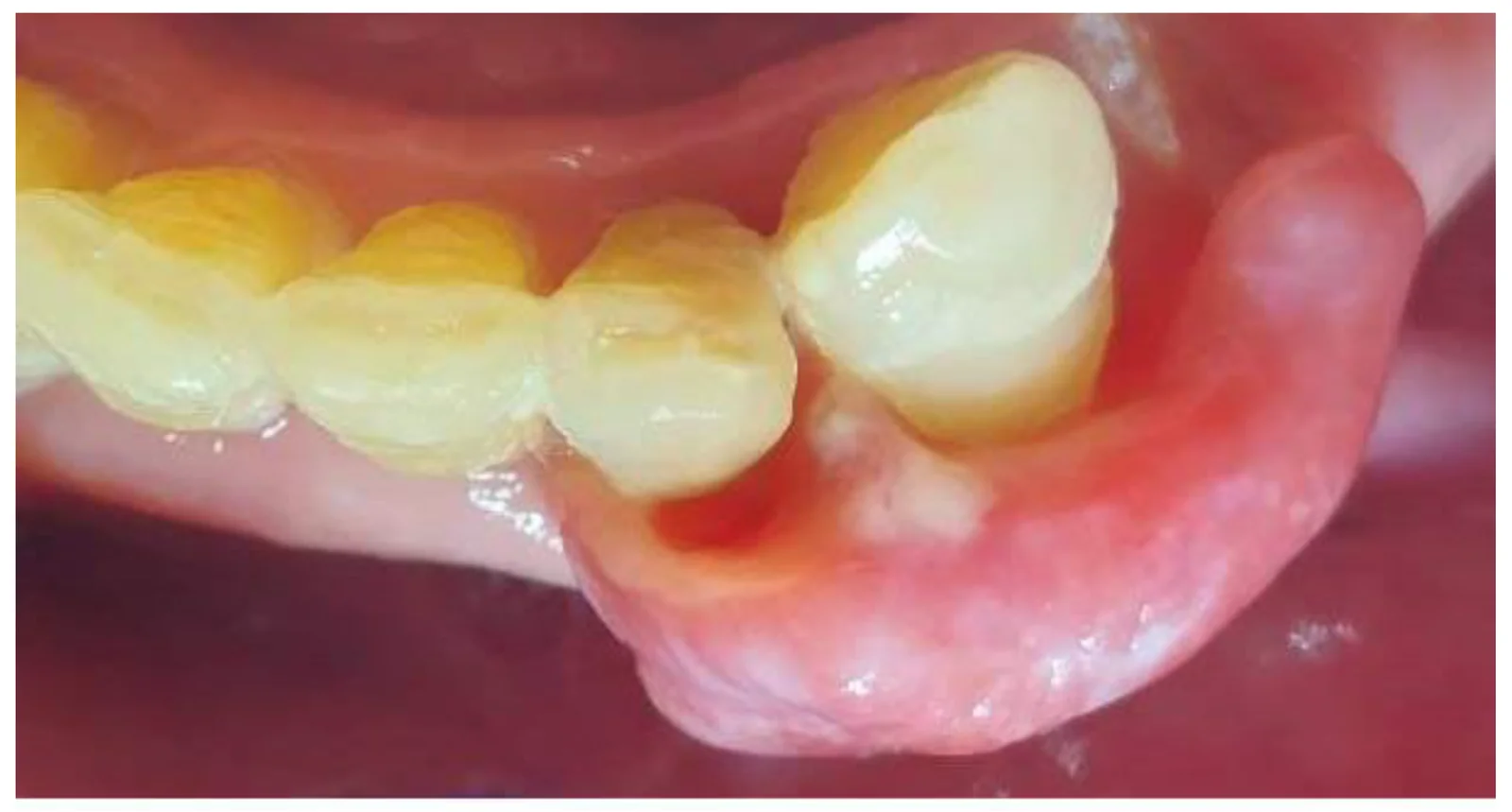
Mandibular tumor in the area of teeth 32–33.

**Figure 3:**
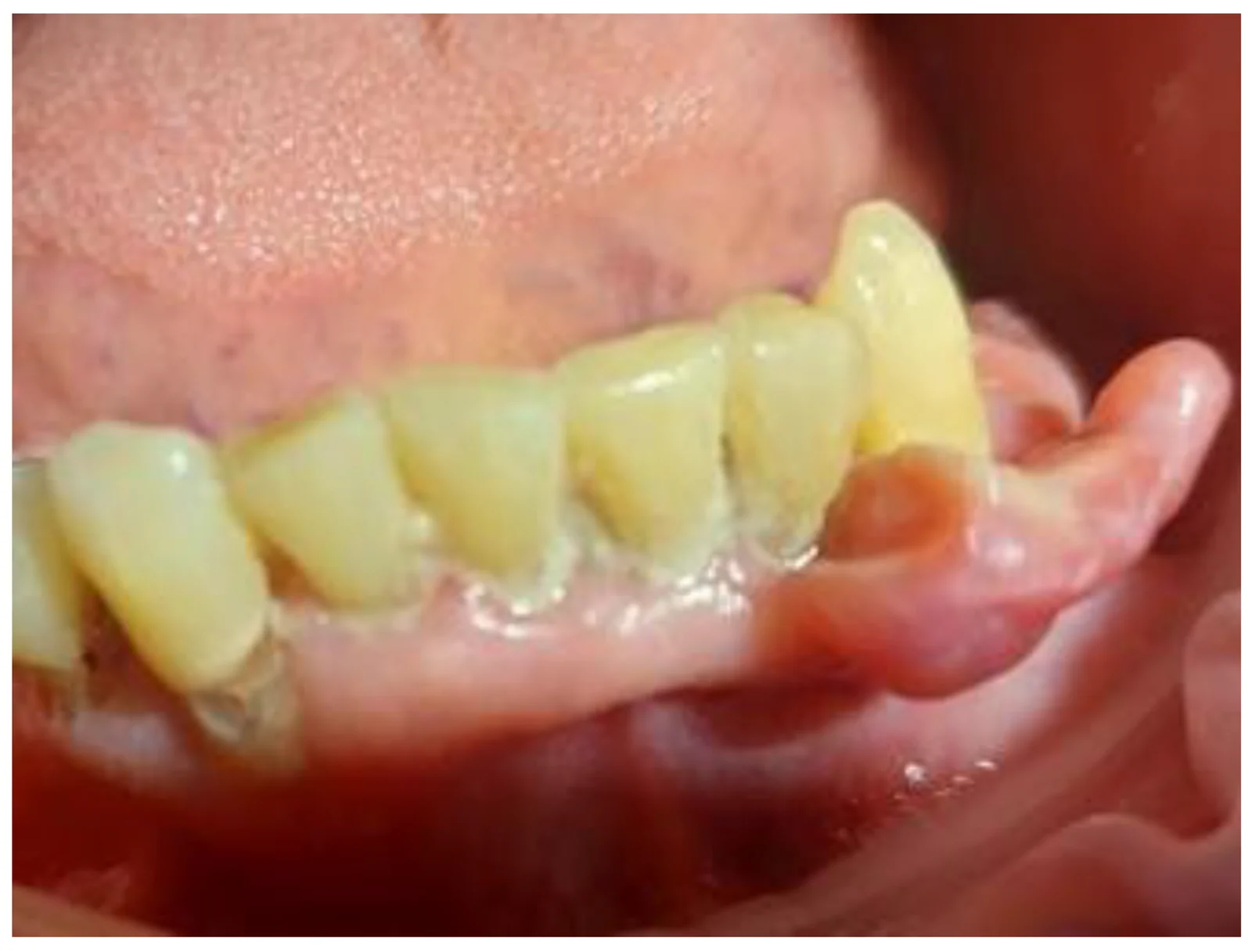
Mandibular tumor in the area of teeth 32–33.

On the day of presentation to the Department of Oral Surgery, extraoral examination revealed painless, enlarged left submandibular lymph nodes. Intraorally, tumor-like gingival proliferations were observed in the regions of teeth 17–16 (22 × 10 × 5 mm) (Figure 1a) and 32–33 (30 × 20 × 6 mm), length, width, and thickness, respectively (Figure 2 and Figure 3). The lesions were pedunculated, elastic-hard, irregularly elliptical, and pink with areas of hyperemia and white spots. Both originated from marginal gingiva and were in close proximity to teeth. The patient also exhibited generalized periodontal disease with grade II mobility of teeth 17, 16, and 33. Residual roots of teeth 34, 35 were present. A panoramic radiograph showed no bony abnormalities. At this stage, the clinical appearance of the gingival lesions was suggestive of reactive hyperplastic conditions such as fibrous hyperplasia or pyogenic granuloma. However, these were considered only preliminary differential diagnoses, with the final diagnosis dependent on histopathological evaluation. The patient provided informed written consent for the proposed treatment.

After oral hygiene treatment, under local anesthesia (articaine and norepinephrine 1:200,000 (40 mg + 0.005 mg) diagnostic excisional biopsies were performed. Both gingival lesions were excised with simultaneous extraction of adjacent teeth. Sharp bone edges were smoothed and the wounds sutured. Specimens were submitted for histopathological evaluation. At one-week follow-up, the sutures were removed (Figure 1b). Healing was uneventful, and the patient reported no complaints.

**Figure 4:**
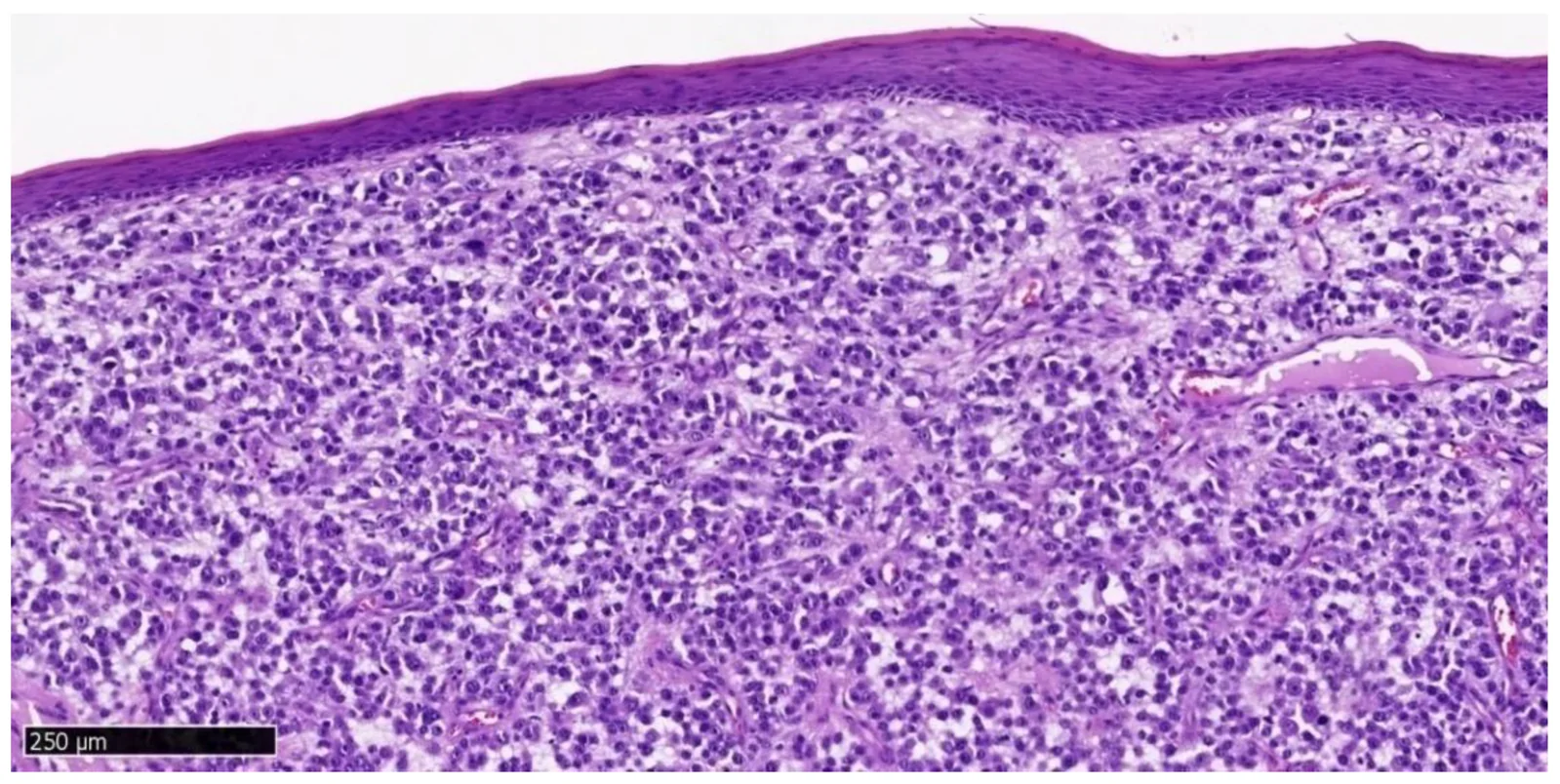
Hematoxylin and eosin staining, final magnification ×100, specimen from the upper gingival tumor. Scale bar: 250 μm.

**Figure 5:**
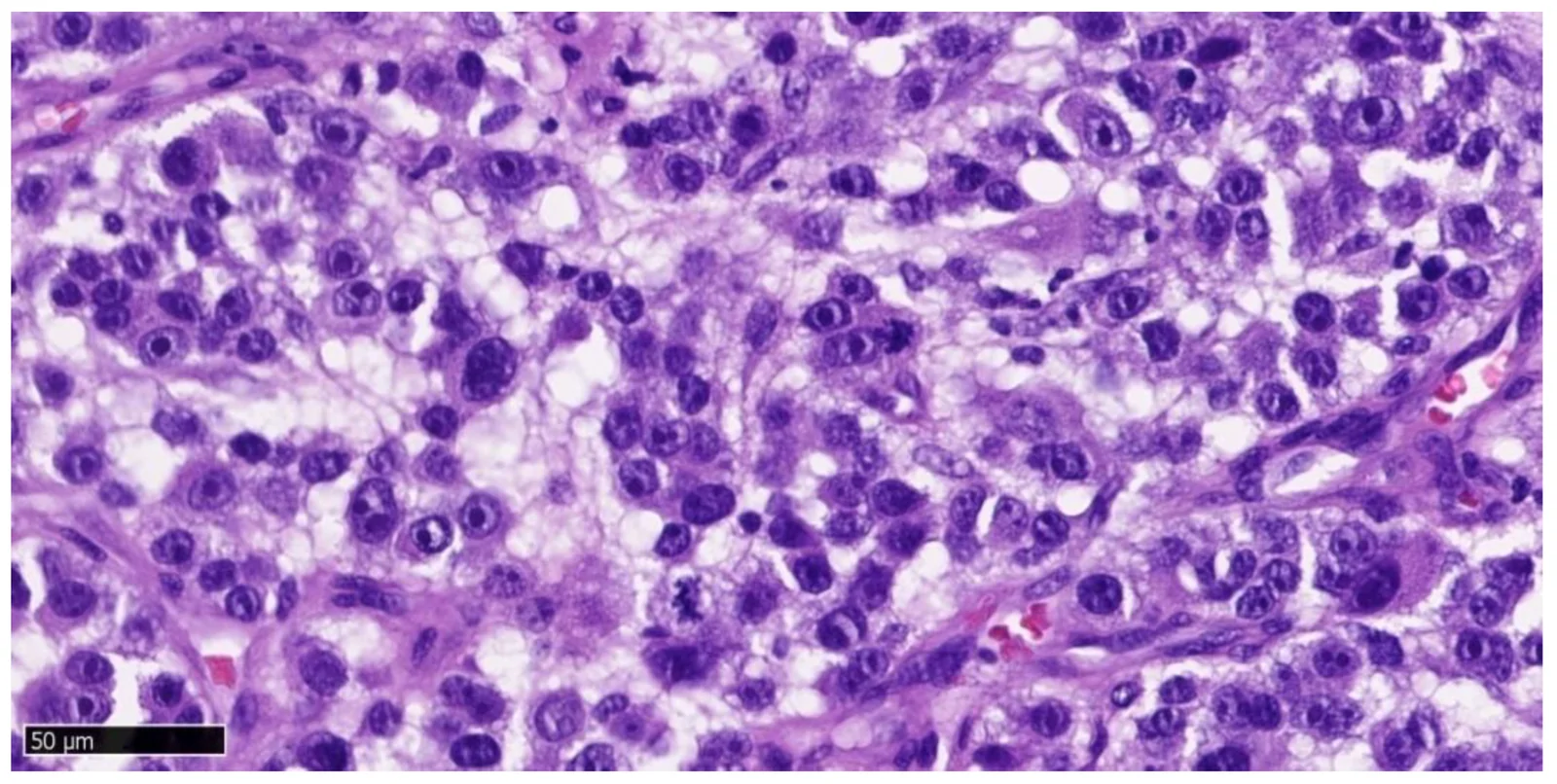
Hematoxylin and eosin staining, final magnification ×400, specimen from the upper gingival tumor. Scale bar: 50 μm.

**Figure 6:**
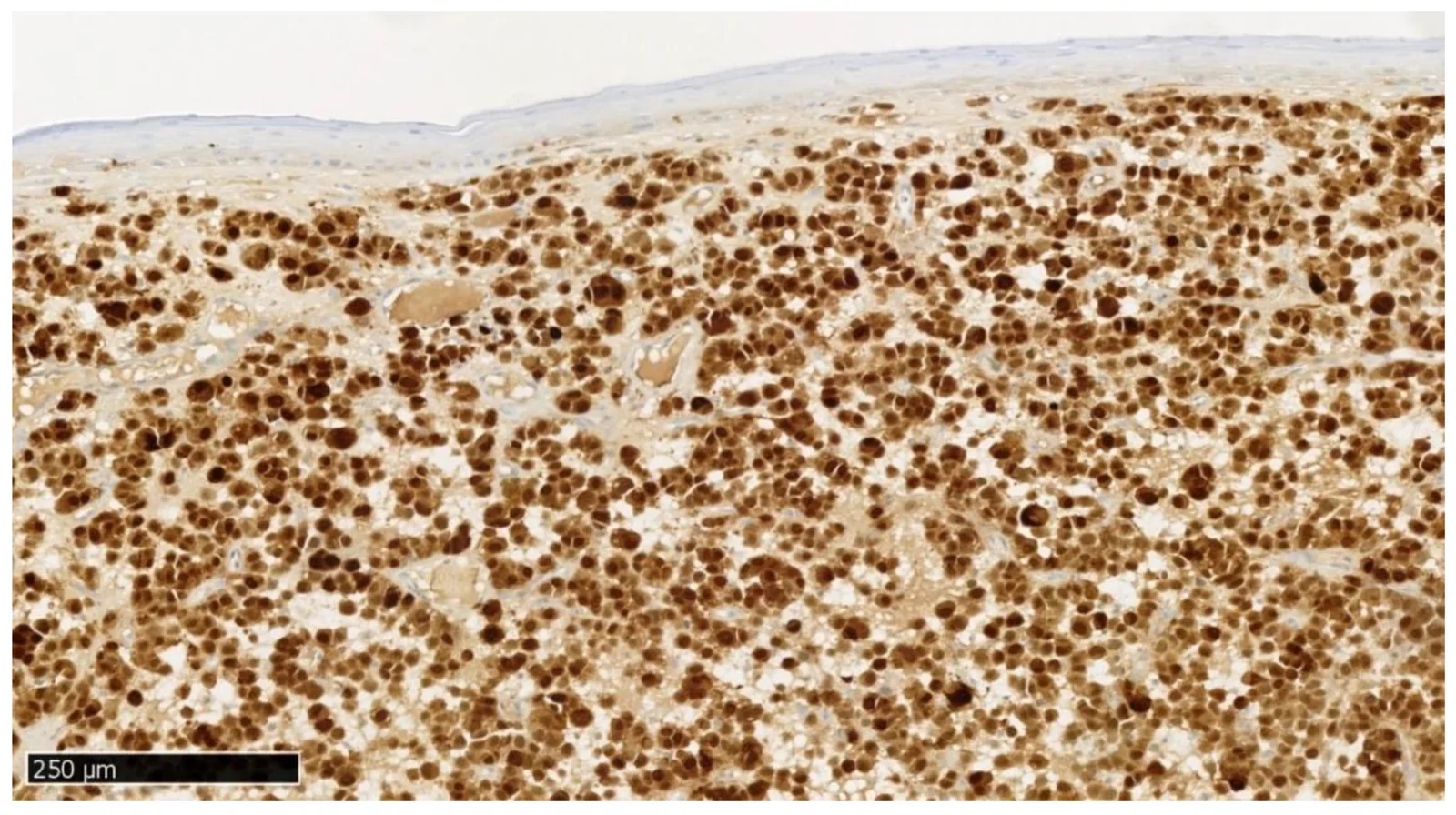
Immunohistochemical staining S100, final magnification ×100, specimen from the upper gingival tumor. Scale bar: 250 μm.

Microscopic and immunohistochemical analysis confirmed the diagnosis of multifocal oral mucosal melanoma (OMM):

Maxillary lesion: infiltrating melanoma, mitotic index 14/mm^2^, ulceration present, margins involved (Figure 4, Figure 5 and Figure 6).

**Figure 7:**
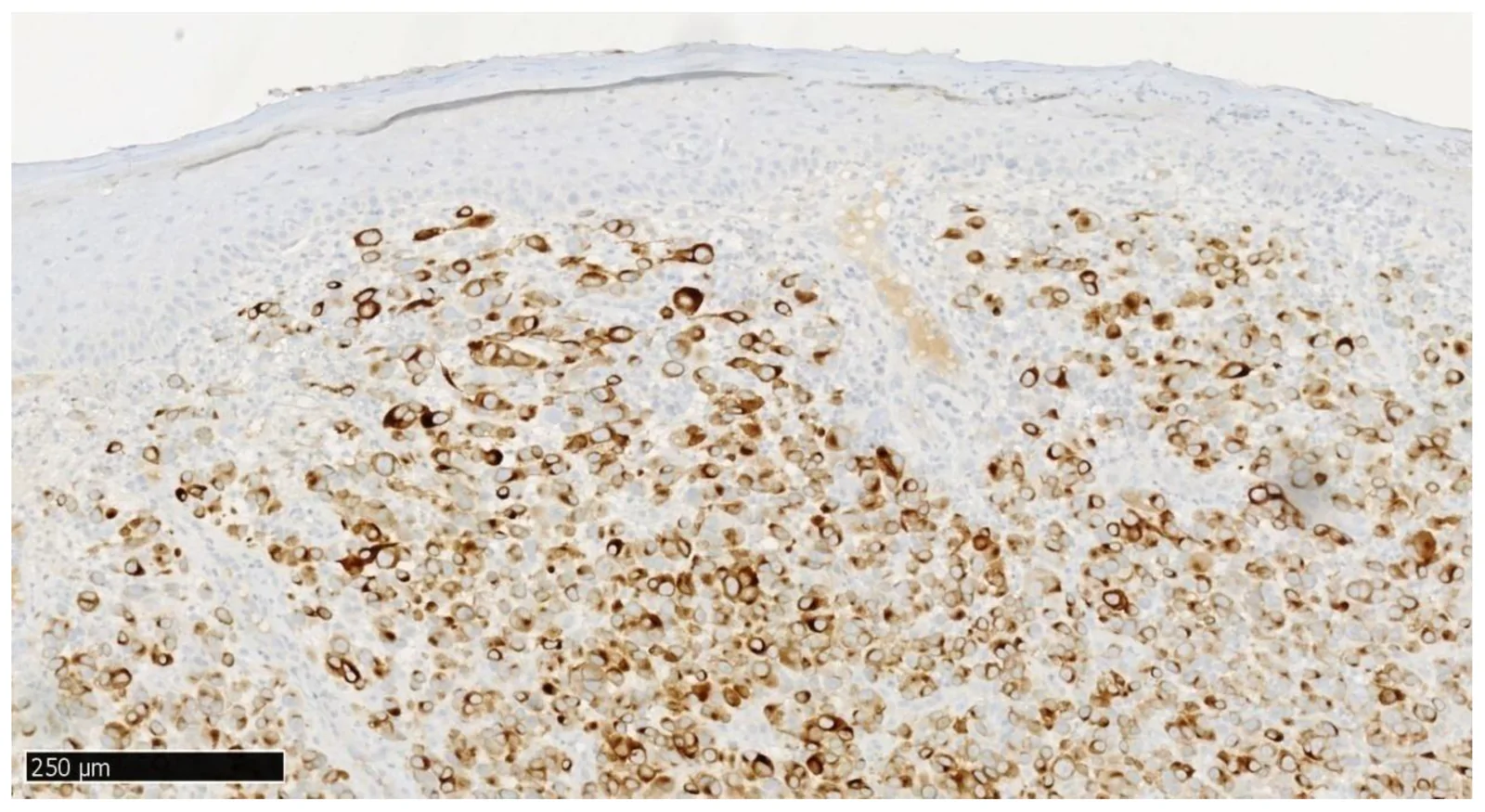
Immunohistochemical staining MelanA/MART1, final magnification ×100, specimen from the lower gingival tumor. Scale bar: 250 μm.

**Figure 8:**
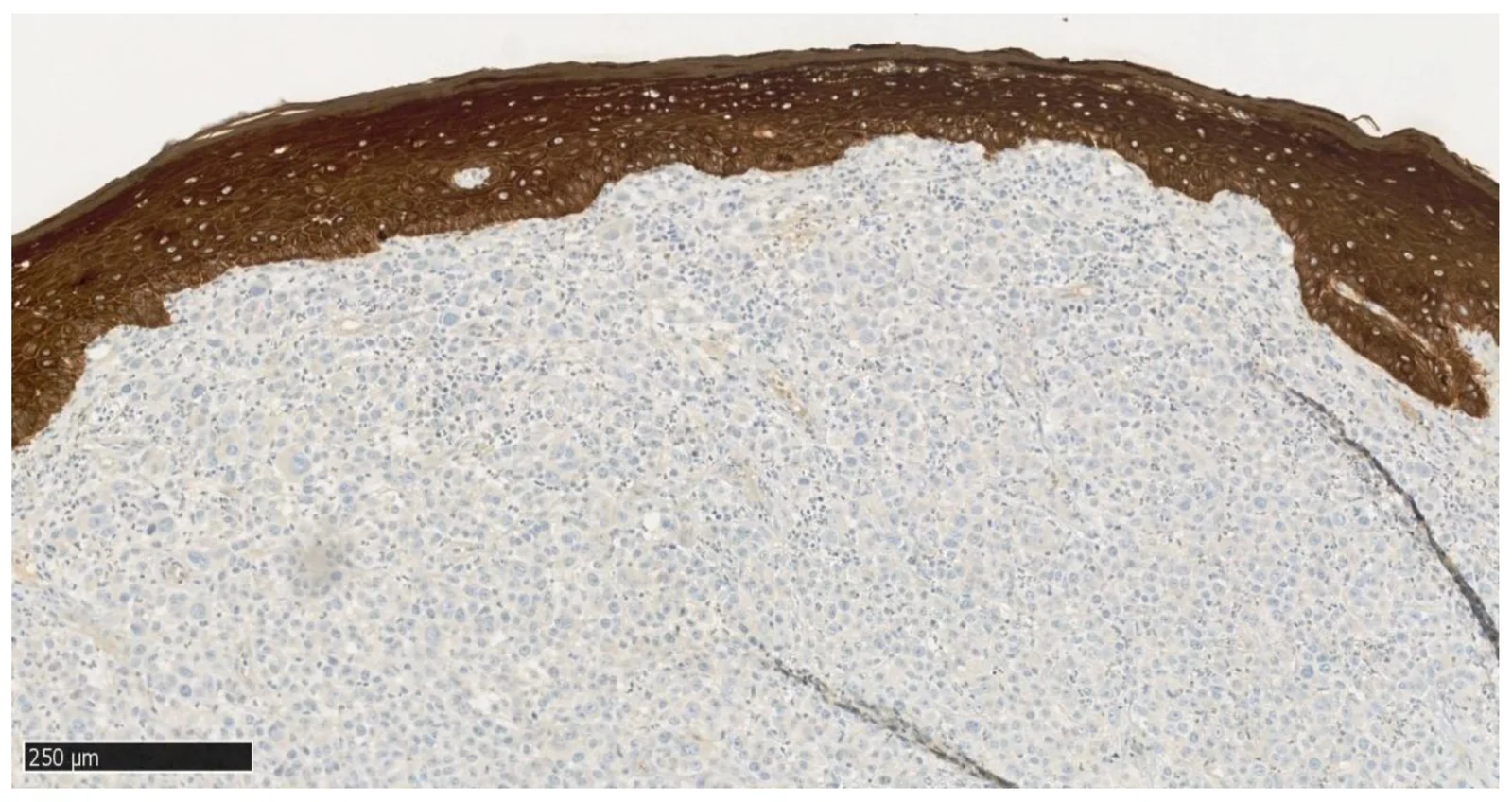
Immunohistochemical staining CKAE1/AE3, final magnification ×100, specimen from the lower gingival tumor. Scale bar: 250 μm.

**Figure 9:**
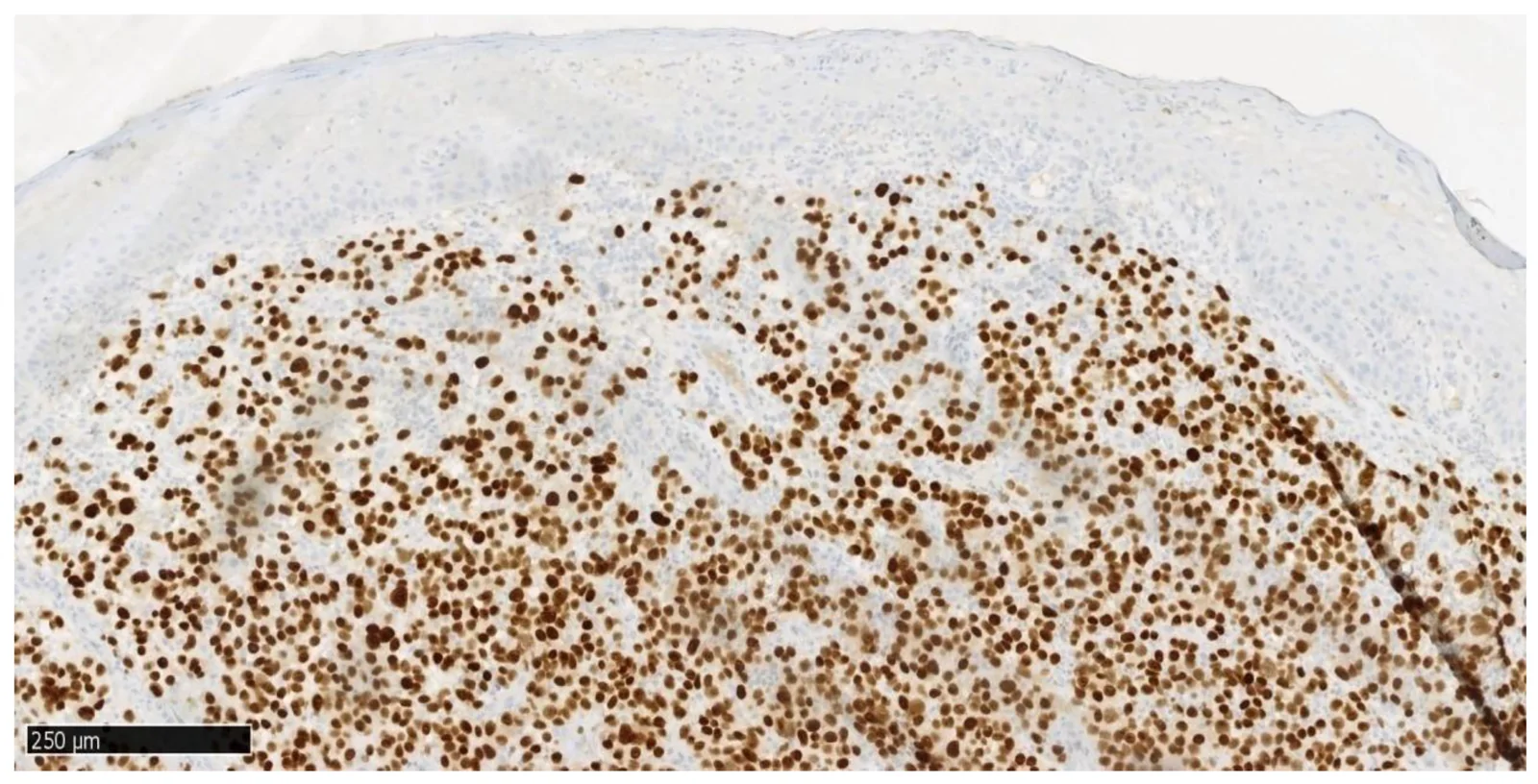
Immunohistochemical staining SOX10, final magnification ×100, specimen from the lower gingival tumor. Scale bar: 250 μm.

**Figure 10:**
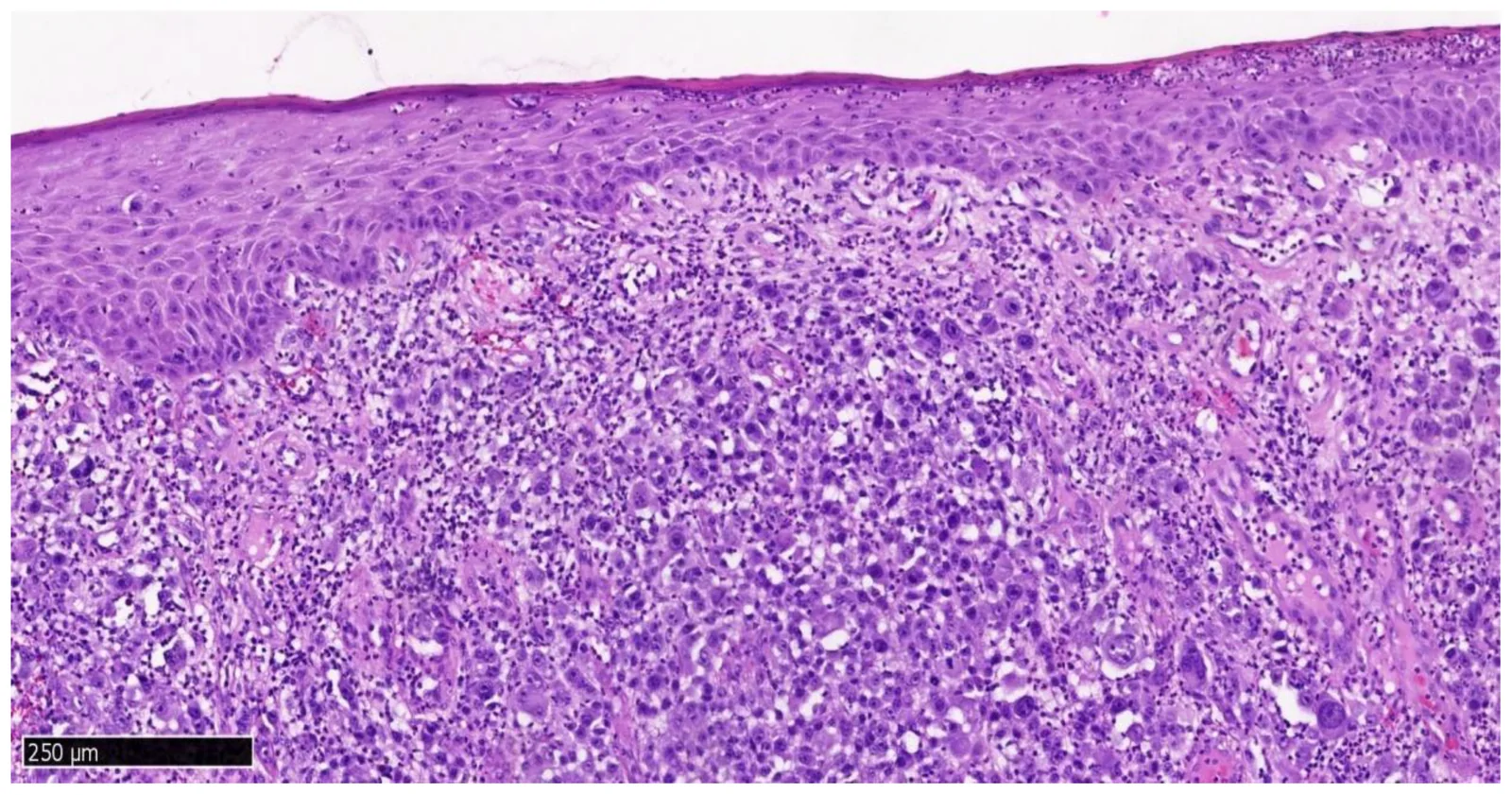
Hematoxylin and eosin staining, final magnification ×100, specimen from the lower gingival tumor. Scale bar: 250 μm.

Mandibular lesion: infiltrating melanoma, mitotic index 5/mm^2^, ulceration present, margins involved (Figure 7, Figure 8, Figure 9 and Figure 10).

Both specimens: non-brisk TILs, no satellite nodules, angioinvasion, or neuroinvasion.

Immunohistochemistry: S100+, SOX10+, MelanA+, Ki67 high (80% positive in maxilla specimen, 70% positive in mandibular specimen); CK Pan−, LCA−.

S100 and SOX10 staining demonstrated strong positivity in melanoma cells with negative reaction in adjacent epithelium. MelanA/MART1 also showed cytoplasmic positivity in tumor cells, whereas CKAE1/AE3 staining was negative in melanoma cells but positive in normal epithelium (Figure 6, Figure 7, Figure 8 and Figure 9). BRAF mutation negative (There is no information in medical history if the KIT and RNAS mutation was tested.)

Histopathological and immunohistochemical analysis confirmed the diagnosis of multifocal oral mucosal melanoma (OMM). According to the AJCC classification of mucosal melanomas of the head and neck [8], the presence of multiple, independent oral mucosal lesions should be interpreted as primary multifocal disease rather than secondary spread. This interpretation is consistent with the WHO Classification of Head and Neck Tumours [9], which recognizes the possibility of synchronous multifocal primary melanomas within the oral cavity. Similar considerations have been highlighted in clinical series, where multifocal OMM has been proposed as a distinct clinicopathological entity requiring precise staging and follow-up [10].

Following histopathological confirmation, the patient was urgently referred to the Oncology Center. Two months later, a local recurrence was observed in the maxillary surgical scar. Contrast-enhanced CT showed a 12 mm nodular thickening in the right upper gingiva, without evidence of lymphadenopathy, bone invasion, or distant metastases. Because of advanced age and high anesthetic risk, the patient declined further surgery and underwent radiotherapy VMAT-CBCT (Volumetric Modulated Arc Therapy-Cone Beam Computed Tomography)—6 Gy/fraction, total dose 30 Gy. The treatment resulted in tumor regression. At three-month follow-up, no recurrence was detected. The patient remains under continuous oncological surveillance.

## 3. Discussion

This case highlights the diagnostic complexity of oral mucosal melanoma (OMM), particularly its amelanotic variant, which commonly presents without characteristic pigmentation and may be mistaken for benign lesions. In our patient, the lesion’s smooth borders, lack of pigmentation, and atypical gingival location deviated from the more common palatal presentation, contributing to diagnostic delay [23]. This underscores the essential role of early biopsy for accurate diagnosis and timely intervention [24,25].

OMM is rare, accounting for 0.2–8% of all melanomas and <2% of oral malignancies [1,26]. It most often affects patients > 50 years old, with higher prevalence in Asian and African populations [15]. Palate and gingiva are the most common locations, with gingival origin considered less typical. Prognosis remains poor, with 5-year survival rates below 25%, largely due to delayed diagnosis and early metastasis [1,2,27].

Oral mucosal melanoma develops asymptomatically but progresses rapidly. Early lesions are flat and small; later they become irregular, pigmented or amelanotic, and ulcerated. Symptoms may include bleeding, tooth mobility, and lymphadenopathy [2,28,29].

Our case stands out in the field of OMM because of its synchronous, multifocal presentation, a rare phenomenon scarcely described in the recent literature. We interpret the oral lesions as synchronous multifocal primary OMM. Contiguous extension refers to the direct radial spread of a single neoplasm across anatomically adjacent mucosal surfaces, whereas multifocality denotes the presence of spatially distinct, histogenetically independent primary tumors. In mucosal melanoma of the head and neck, the occurrence of multiple foci lies within a diagnostic gray zone, as such lesions may reflect contiguous local progression, a mucosal field effect, or the emergence of de novo independent primaries. In the present case, the presence of well-demarcated, non-contiguous oral sites without intervening mucosal involvement most plausibly supports the designation of synchronous multifocal primaries. The amelanotic nature of the lesions significantly impedes clinical recognition, making histopathological examination and immunohistochemical profiling indispensable. Amelanotic OMM presents specific diagnostic challenges. Lack of pigmentation often leads to misdiagnoses and treatment delays. In a series of eight OAM (oral amelanotic melanomas), the majority presented as ulcerated masses, were located on gingiva or palate, and carried extremely poor prognoses; in fact, most patients succumbed within months of diagnosis [30]. Other reports highlight that amelanotic melanoma can mimic a broad spectrum of pathologies—ranging from squamous cell carcinoma, sarcoma, to poorly differentiated carcinoma—necessitating a thorough immunohistochemical approach for accurate diagnosis [31].

In the differential diagnosis of amelanotic melanoma, Kaposi’s sarcoma, pyogenic granuloma, fibrous hyperplasia, salivary gland tumors, lymphomas and metastasis should be considered. If the pigmentation of a lesion cannot be definitively diagnosed as benign based on clinical findings, a biopsy is mandatory to exclude OMM. Inflammatory fibrous hyperplasia (IFH) is a common reactive lesion of the oral mucosa. Clinically, it often presents as a well-defined exophytic mass with firm consistency and normal gingival coloration. On palpation, it may be either soft or hard.

These atypical features necessitate heightened clinical suspicion, prompt diagnostic work-up, and tailored staging and management strategies, reinforcing that such rare presentations warrant special attention in both clinical practice and future research.

A multidisciplinary team (MDT) approach was considered essential for the management of this patient. Following histopathological confirmation of multifocal amelanotic oral mucosal melanoma, the case was reviewed by the tumor board, including specialists in oral and maxillofacial surgery, oncology, radiology, radiation therapy, and pathology. The MDT discussion guided the decision to prioritize radiotherapy over surgery due to the patient’s advanced age, comorbidities, and high anesthetic risk. A structured follow-up schedule was established, with clinical and imaging evaluations at 3-month intervals to monitor for recurrence or metastasis, ensuring comprehensive surveillance despite the patient’s limitations.

From a dental perspective, this case underscores the responsibility of dentists and oral surgeons to recognize and appropriately manage atypical oral lesions. Gingival overgrowths are frequently interpreted as inflammatory or reactive in origin; however, this report illustrates that persistent or unusual lesions warrant histopathological evaluation to rule out serious conditions such as OMM [25,26]. For this reason an algorithm of management for general dentists has been proposed (Table 1).

Histologically, amelanotic oral mucosal melanoma consists of spindle cells with numerous mitotic figures and no cytoplasmic melanin pigmentation. These malignant tumor cells are characterized by significant pleomorphism, with large, irregular, hyperchromatic nuclei and prominent nucleoli [18]. The cells of malignant oral mucosal melanoma can be visualized using hematoxylin and eosin staining. However, if melanin is absent in amelanotic melanoma, immunohistochemical (IHC) staining should be performed to obtain additional diagnostic information. CK and LCA staining are used for differential diagnosis with squamous cell carcinoma and lymphoma. The IHC panel in our case (SOX10/S100 positive; CK Pan negative; variable melanocytic marker expression) aligns with recommended diagnostic strategies for OMM. Specifically, SOX10 has demonstrated greater expression stability compared to S100 in melanoma diagnostics, while Melan-A may be negative in amelanotic variants, highlighting the need for panel-based interpretation rather than reliance on individual markers [17,32]. Moreover, a very high Ki-67 index (70–80% in our case) and elevated mitotic index (MI: upper gum tumor—14/mm^2^; lower tumor—5/mm^2^) correspond with more aggressive tumor biology and poorer prognosis in head and neck OMM. High Ki-67 proliferation indices (60–100%) have been correlated with shorter overall survival in mucosal melanoma patients [33,34].

Sometimes, satellite nodules may occur, i.e., nests of tumor cells separated from the main lesion. These are more common in cutaneous melanoma than in mucosal lesions, and their presence is associated with worse prognosis [22]. Lymphocytic infiltrates in oral melanoma reflect the body’s immune response to the developing tumor. The presence of these infiltrates, known as tumor-infiltrating lymphocytes (TILs), can be used to predict patient prognosis. Dense peritumoral lymphocytic infiltrate is associated with a better prognosis. In our patient, lymphocytic infiltrates were present and were described as moderately abundant (non-brisk). In the histopathological evaluation of oral mucosal melanoma, the depth of infiltration is not assessed using the Clark or Breslow scales, as these methods were developed specifically for cutaneous melanoma. The structural differences between oral mucosa and skin—particularly its reduced thickness and the absence of distinct histological landmarks, such as the papillary and reticular dermal layers—render these scales inapplicable [14]. Moreover, studies have shown that the depth of invasion does not significantly correlate with prognosis in mucosal melanoma [32]. Instead, the presence of vascular invasion is considered a critical adverse prognostic factor, as intravascular access facilitates dissemination via lymphatic and hematogenous routes. Ultimately, the development of distant metastases remains the single most important determinant of survival in patients with these tumors [2].

Histopathological examination confirmed oral mucosal melanoma but does not determine whether the lesion is primary or metastatic. Traditional histopathologic features, such as junctional activity, are now considered unreliable because metastatic lesions may exhibit similar patterns. None of the examined samples showed an in situ or junctional component. Although no other primary melanoma was detected in our patient, confirming the origin (primary vs. metastatic) relies on molecular and genetic studies, which remain largely investigational and provide limited clinical guidance [5,17,25].

Computed tomography (CT) with contrast is used to assess the primary tumor and cervical lymph nodes, and can also detect bone lesions. Magnetic resonance imaging (MRI) is employed to evaluate melanoma in soft tissues and is particularly useful for assessing the extent of tumors in the paranasal sinuses, especially those involving the skull base or demonstrating neurotropic spread. Tests for detecting distant metastases include brain MRI, chest CT, bone scintigraphy, and/or positron emission tomography (PET) [35].

The 18F-FDG PET/CT protocol (positron emission tomography integrated with computed tomography using the glucose radioisotope fluorodeoxyglucose—FDG) has demonstrated high diagnostic accuracy in detecting metastases in soft tissues, lymph nodes, and internal organs both at initial assessment and during follow-up. It can also detect early tumor response during tyrosine kinase inhibitor (TKI) treatment [36,37]. The primary treatment for patients with oral mucosal melanoma is radical surgical excision within healthy tissue margins. Guidelines for the surgical management of primary cutaneous melanoma recommend a diagnostic excisional biopsy, followed by excision of the scar with an appropriately wide margin of macroscopically normal tissue after diagnosis. In the oral cavity, however, lesion size and anatomical limitations may complicate the performance of an excisional biopsy. Recommendations for surgical margins vary between 1.5 and 2.5 cm, but no consensus guidelines exist for OMM [4,6]. According to the guidelines of the National Comprehensive Cancer Network (NCCN, USA), primary treatment should be surgical for stage III to IVA according to the AJCC TNM classification (Table S1), whereas surgery is not recommended in stages IVB and IVC; such patients should be referred for clinical trials or radiotherapy [34]. Radiotherapy can improve local control but does not significantly affect overall survival [38]. Novel therapeutic approaches include immunotherapy and targeted therapy (BRAF/MEK or KIT inhibitors), although mutation profiles differ from cutaneous melanoma, with KIT and NRAS mutations more common than BRAF [1,27].

In our patient, the BRAF V600 mutation was not detected, precluding targeted therapy. Testing for KIT, NRAS, and NF1 mutations, which could have been performed on paraffin-embedded tissue using targeted next-generation sequencing (NGS), was not undertaken due to the patient’s advanced age, poor general condition, low Eastern Cooperative Oncology Group (ECOG) performance status, and personal preferences, which could represent contraindications to immunotherapy. This is particularly relevant in OMM, where mutation rates differ significantly from cutaneous melanoma; for instance, KIT mutations may occur in up to 25% of cases, while BRAF mutations are uncommon (<6%) [27]. In rare and aggressive tumors such as OMM, discussion within a multidisciplinary tumor board (MDT) and consideration of personalized oncology approaches are crucial. NGS (next-generation sequencing) plays a central role in this context: it enables broad genomic profiling from small FFPE (formalin-fixed, paraffin-embedded) samples, refines diagnosis and tumor lineage, assesses clonality, uncovers actionable alterations, informs prognosis, and matches patients to relevant clinical trials when standard tests and evidence are limited. Thus, incorporating NGS and personalized therapeutic strategies may improve patient stratification and expand treatment options even in tumors with limited standard-of-care pathways [39,40]. In our case, however, these options were not pursued due to the clinical constraints described above. Additionally, PET/CT imaging was not performed, and contrast-enhanced CT was used for staging instead. This decision was based on limited PET/CT availability, patient preference, and a low pretest probability of metastatic disease. However, substituting PET/CT with CT may reduce sensitivity for detecting small-volume nodal or distant metastases, thereby affecting certainty regarding stage assignment and prognosis. This limitation must be considered when interpreting the results and comparing them with PET/CT-based reports [41,42].

Despite these constraints, the case highlights the necessity of oncological vigilance in dental settings. It broadens awareness of OMM’s diverse clinical presentations and reinforces the importance of early biopsy in atypical cases [26,43,44]. Furthermore, this case contributes to the understanding of the biologic behavior of oral melanoma, emphasizing the need for future reports that include extended molecular profiling to clarify pathogenesis and advance targeted treatment strategies [26,27].

## 4. Conclusions

Oral mucosal melanoma is a rare but aggressive malignancy that presents significant diagnostic challenges, particularly in its amelanotic form. Any atypical or proliferative oral lesion—especially in patients with a history of melanoma—should be biopsied to exclude recurrence or metastasis. Early diagnosis and radical excision remain crucial for improving outcomes, while lifelong surveillance is essential due to the high risk of recurrence. This case underscores the importance of oncological vigilance in dental practice and the need to include mucosal melanoma in the differential diagnosis of gingival and other oral lesions.

## Figures and Tables

**Table 1:** Diagnostic Algorithm for Pigmented/Atypical Oral Lesions (based on generally accepted clinical standards in dentistry and oral oncology as well as literature data).

Step 1: Initial Clinical Assessment	
	Assess lesion characteristics: color, size, shape, surface, symmetry, borders, growth rate
	Identify symptoms: pain, bleeding, ulceration.
	Review patient history: trauma, systemic disease, medications, previous malignancies.
Step 2: Risk Stratification	
	Low-risk features: small, stable, uniform color, smooth surface, asymptomatic → monitor and document.
	High-risk features: rapidly growing, irregular shape/borders, multiple colors, ulceration, bleeding, persistent > 2 weeks, previous melanoma → proceed to biopsy.
Step 3: Basic Investigations	
	Intraoral photographs.
	Radiographs if bone involvement suspected.
	Consider initial lab tests if systemic disease suspected.
Step 4: Biopsy Decision	
	Indicated for any high-risk lesion or atypical presentation.
	Type of biopsy: excisional if small, incisional if large or anatomically challenging.
	Ensure adequate margins for histopathological assessment.
Step 5: Histopathological and Immunohistochemical Analysis	
	Standard H&E staining.
	Immunohistochemistry: S100, SOX10, HMB45, Melan-A, Ki-67.
	Molecular testing (KIT, NRAS, BRAF) if clinically relevant.
Step 6: Multidisciplinary Team (MDT) Review	
	Pathologist, oral/maxillofacial surgeon, oncologist, radiotherapist.
	Decide on definitive treatment and follow-up schedule.
Step 7: Follow-Up	
	Clinical examination every 3–6 months.
	Imaging (CT/MRI) as indicated.
	Educate patient on self-monitoring for new lesions.

## Data Availability

The original contributions presented in this study are included in the article/Supplementary Material. Further inquiries can be directed to the corresponding author.

## Supplementary Materials

The following supporting information can be downloaded at: https://www.mdpi.com/article/10.3390/dj13090432/s1, Table S1: AJCC TNM clinical classification according to TNM staging AJCC UICC 8th edition.
