# Supplementary material for: Multifocal Oral Mucosal Melanoma with an Atypical Clinical Presentation
Source: Dent J (Basel). 2025 Sep 18;13(9):432. doi: 10.3390/dj13090432 (PMC12468697; doi:10.3390/dj13090432)
Supplement: Supplementary file 1 [file dentistry-13-00432-s001.zip › dentistry-3801945-supplementary.pdf]

**Table S1.** AJCC TNM clinical classification according to TNM staging AJCC UICC 8th edition.

| Category | Definition |
|---|---|
| **Primary Tumor (T)** | |
| TX | Primary tumor cannot be assessed |
| T0 | No evidence of primary tumor |
| T3 | Lesions limited to the mucosa and soft tissues directly beneath it, regardless of thickness or largest dimension (e.g., multifocal lesions of the nasal cavity, pigmented or non-pigmented lesions of the oral cavity, pharynx, or larynx) |
| T4a | Moderately advanced lesions. Deep infiltration into soft tissues, cartilage, bone, or skin covering |
| T4b | Very advanced lesions. Involvement of brain, dura mater, skull base, cranial nerves (IX–XII), carotid artery, prevertebral space, or mediastinal structures |
| **Regional Lymph Nodes (N)** | |
| NX | Regional lymph nodes cannot be assessed |
| N0 | No regional lymph node metastases |
| N1 | Metastases to regional lymph nodes |
| **Distant Metastasis (M)** | |
| M0 | No distant metastases |
| M1 | Distant metastases present |
| **Stage Grouping** | |
| Stage III | T3 N0 M0 |
| Stage IVA | T4a N0 M0; T3/T4a N1 M0 |
| Stage IVB | T4b, any N, M0 |
| Stage IVC | Any T, any N, M1 |
